# No Time Like the Present: Thinking About the Past and the Future Is Related to State Dissociation Among Individuals With High Levels of Psychopathological Symptoms

**DOI:** 10.3389/fpsyg.2018.02465

**Published:** 2018-12-07

**Authors:** Miriam Vannikov-Lugassi, Nirit Soffer-Dudek

**Affiliations:** Consciousness and Psychopathology Laboratory, Department of Psychology, Ben-Gurion University of the Negev, Beersheba, Israel

**Keywords:** dissociation, sleep, stress, rumination, worry, absorption, depersonalization-derealization, mind-wandering

## Abstract

Several short-term pathways have been implicated in relation to dissociative experiences, among them are daily stress, sleepiness, and rumination. In addition, it has been claimed that mechanisms contributing to dissociative experiences may differ, according to specific psychopathological symptoms. Accordingly, this study had two aims. The first was to sample moment-to-moment increases or decreases in current stress, sleepiness, and rumination, in order to assess their temporal relations with state dissociation. Rumination was broken down to its basic two subcomponents: the negative value of the thoughts and thinking about the past (in comparison to present or future), in order to differentiate it from other repetitive thought patterns (e.g., worry). The second goal was to explore whether depression, anxiety, and obsessive–compulsive symptoms may moderate the links between the three mechanisms and specific state dissociation scales [specifically, depersonalization-derealization (DEP-DER) and absorption (ABS)]. Ninety-nine undergraduate students completed trait questionnaires and then answered state items four times a day for 4 days. These experience sampling data were analyzed using multilevel linear modeling (MLM) with Level 1 state measurements and Level 2 demographic and trait variables of the participants. Moments of stress, sleepiness, thinking about the past and negative thoughts were all associated both with state DEP-DER and with state ABS. Dissociation, negative thinking, stress, and sleepiness were positively associated with moments of thinking about the past and the future but inversely associated with moments of thinking about the present. Finally, in accordance with our expectations, the links between DEP-DER and hypothesized mechanisms were mostly moderated by depression and anxiety symptoms, whereas the links between ABS and hypothesized mechanisms were moderated mainly by obsessive–compulsive symptoms. Our findings are in accordance with literature on the efficacy of mindfulness as well as the maladaptive correlates of mind-wandering, as they suggest that dissociative detachment from one’s present occupation is associated with decreased well-being.

## Introduction

Dissociation is a disruption of the normal integration of consciousness (DSM-5, American Psychiatric Association, 2013), i.e., a discontinuity between mental contents (e.g., thoughts, feelings, or memories). The tendency to dissociate is maladaptive; it is related to the experience of psychological distress (e.g., Gershuny and Thayer, 1999; Briere et al., 2005b; Dalenberg et al., 2012; Soffer-Dudek, 2014). Dissociation is associated with several psychopathological symptoms, such as post-traumatic stress, depression, anxiety, eating disorders, and obsessive–compulsive (OC) symptoms (e.g., Wise et al., 2000; Muris et al., 2003; Lipsanen et al., 2004; Watson et al., 2004; Pastucha et al., 2009; Paradisis et al., 2015; Soffer-Dudek, 2017a), as well as with poorer treatment outcomes (Rufer et al., 2006; Spitzer et al., 2007; Fassino et al., 2009; Semiz et al., 2014; Arntz et al., 2015). Thus, it is important to understand specific daily variables that may be associated with increases in dissociative experiences, and their mechanisms of interaction with various types of psychological distress symptoms (Soffer-Dudek, 2014). The present study aimed to promote such understanding by employing a rigorous experience-sampling methodology exploring these short-term dynamics with a fine-grain resolution. In this study, we focused on three factors that vary from one moment to the next and have all been associated with dissociative experiences, specifically, current stress, sleepiness, and rumination. As will be described in detail below, we further explore rumination, and differentiate it from worry, by breaking it down to its basic components. Thus, we also explore correlates of thinking about the past, the present, or the future, in this experience-sampling study.

### Factors Related to Dissociative Experiences: Daily Stress, Sleepiness, and Rumination

Many researchers consider trauma to be the origin of dissociation. Pierre Janet was the first to identify dissociation as a defense or coping mechanism in the face of overwhelming emotion (Van der Hart and Horst, 1989), and later theoreticians (e.g., Putnam, 1991, 1996) expanded this view. They suggest that dissociation enables mental escape from distress by denying introspective access to unbearable mental contents. Importantly, this coping strategy is considered to be maladaptive because it interferes with adaptive emotional processing of distressing experiences (Spiegel, 1991; Foa and Hearst-Ikeda, 1996; Schauer and Elbert, 2010). In accordance with this theory, trauma is related to dissociation (e.g., Morgan et al., 2001; Carlson et al., 2012, 2016; Dalenberg et al., 2012; Terock et al., 2016). Overwhelming anxiety in the form of a panic attack is also often accompanied by dissociation (Hunter et al., 2004; Mendoza et al., 2011). Researchers have suggested that the tendency to dissociate, or to react with emotional detachment to trauma or to extreme stress, may over time be used non-selectively when confronting even minor stressors (Perry et al., 1995; Spiegel et al., 2011). However, in comparison with trauma or extreme stress, *current daily stress* has not been investigated as much in the context of dissociation. Findings from longitudinal studies show that daily stress is associated with dissociative experiences both among subjects who were exposed to trauma (e.g., Stiglmayr et al., 2008) and non-clinical subjects that were probably not exposed to trauma (Stiglmayr et al., 2008; Soffer-Dudek, 2017a). The aim of this study is to explore the short-term predictors of dissociative experiences and thus we focus on the relationship between momentary stress and dissociation, regardless of possible past traumas that may increase the tendency to dissociate.

Another short-term pathway which has been implicated in relation to dissociative experiences are alterations in the *sleep cycle* (van der Kloet et al., 2012b). Specifically, dissociation may follow impaired sleep patterns, as it appears to be related to a labile sleep–wake cycle and possibly represents intrusions of sleep elements into waking consciousness (Mahowald and Schenck, 2001; Koffel and Watson, 2009; Lynn et al., 2012; van der Kloet et al., 2012b) or waking elements into sleeping consciousness (Soffer-Dudek, 2017b). Indeed, it has been demonstrated that sleep deprivation significantly increases dissociative symptoms (Giesbrecht et al., 2007; Selvi et al., 2015; van Heugten-van der Kloet et al., 2015b; Soffer-Dudek et al., 2017). In addition, practicing sleep hygiene is associated with a reduction of dissociative symptoms in psychiatric inpatients (van der Kloet et al., 2012a).

Recently, poor sleep quality was also identified as the main factor responsible for the link between *rumination* and dissociation (Vannikov-Lugassi and Soffer-Dudek, 2018). Rumination is defined as a repetitive focusing on one’s distress, on sadness, and on the circumstances associated with those feelings, such as their causes and consequences (Nolen-Hoeksema, 1991; Conway et al., 2000). Ruminative repetitive thoughts may also represent goal discrepancy, i.e., the discrepancy between the actual and desired status of achieving one’s goals (Martin and Tesser, 1996). Although the definitions of rumination vary, all refer to the experience of repetitive, intrusive, and negative cognitions (Querstret and Cropley, 2013), and indeed, it plays a role in the development and persistence of negative moods (Smith and Alloy, 2009). Rumination is positively related to dissociation (Armey and Crowther, 2008; Vannikov-Lugassi and Soffer-Dudek, 2018). Notably, however, the studies regarding the link between rumination and dissociation are cross-sectional and may be biased by individual differences in self-reporting patterns. Closely related concepts of thought styles have also been associated with dissociation. For example, dissociation partially mediated the relation between inner speech and self-referential ideas (Bellido-Zanin et al., 2016); such inner speech characterizes rumination, which is related to verbal processes inherent in internal dialog (Nolen-Hoeksema, 2004). In addition, an experience-sampling study conducted on a single clinical case (Poerio et al., 2016) found that repetitive and negative daydreaming by the client predicted increased dissociation. Moreover, Recent evidence suggests that daydreaming may be maladaptive and addictive, and that such maladaptive daydreaming (MD) is related to psychopathological distress and specifically to dissociation (Somer, 2002; Somer et al., 2017; Soffer-Dudek and Somer, 2018). These findings imply that rumination, which is characterized by inner speech (Nolen-Hoeksema, 2004) and by repetitiveness and negative valence (Nolen-Hoeksema, 1991), predicts dissociation. However, they also raise a question regarding the specificity of the effect of ruminative thinking on dissociation, in comparison with closely related types of thought styles.

Watkins (2008) argues that thinking is defined by three factors: the value of the content, the structure of the thoughts, and the context (e.g., mood, beliefs about the self). According to this scheme, rumination may be viewed as possessing a negative content value, including depression, pessimism, and anxiety (e.g., Nolen-Hoeksema, 1991) and a passive and repetitive thought structure. Both repetitiveness and negative emotion were predictors of dissociation, mediated by poor sleep (Vannikov-Lugassi and Soffer-Dudek, 2018), suggesting that both these aspects of rumination play a role in the rumination-dissociation link. Interestingly, the concept of worry is also characterized by negative content and a repetitive structure (Borkovec et al.,1998), and is thus closely related to rumination. In addition, like rumination, worry is related to dissociation (Vannikov-Lugassi and Soffer-Dudek, 2018; Yıldırım et al., 2018). However, despite these similarities, the difference between rumination and worry is in one element of their content: rumination includes focusing on the past, whereas worry is defined by focusing on the future (Borkovec et al., 1998). These similarities between rumination and worry underscore the question regarding the specificity of the effect of rumination on dissociation.

Rumination, as well as worry, is often explored as a whole construct. In order to gain a better understanding of the relations between thought components that associate with dissociation, there is a need for a high-resolution study that may break these concepts down to their foundations (i.e., the basic elements of thought styles) and explore the temporal links between the factors. Hence, in this study, we split the concept of ruminative thought into two components: (a) negative value; and (b) focusing on the past (in comparison to focusing on the future as in worry, or to focusing on the present). Our first aim of the study was to explore the links between dissociation and possible short-term hypothesized predictors: momentary stress, sleepiness, and the components representing rumination, using an experience-sampling method, thus gaining a better understanding of the moment-to-moment mechanisms governing dissociative experiences.

### Differentiating Between Types of Dissociative Experiences

Notably, dissociation is not a homogeneous trait but a multifaceted phenomenon (Briere et al., 2005a), and the presentations of dissociation may include a variety of symptoms (e.g., Holmes et al., 2005). Specifically, Carlson and Putnam (1993) described three empirically derived factors based on factor analyses of the revised version of the Dissociative Experiences Survey (DES-II). *Dissociative amnesia* is a discontinuity between memory and awareness, referring to difficulties in accessing autobiographical memory. *Depersonalization-derealization* (DEP-DER) is an experience of the self or the world as altered or unfamiliar. *Dissociative absorption and imaginative involvement* (ABS) is the tendency to involuntarily narrow attention to the point of obliviousness to the environment (Soffer-Dudek et al., 2015) and it involves a temporary lack of reflective consciousness (Butler, 2006).

Although the general dissociation score is often used when measuring the correlations between dissociation and various emotional states, Soffer-Dudek (2014) suggested that each of the subcomponents of dissociation may play a unique role in different psychopathologies. Specifically, Soffer-Dudek hypothesized that DEP-DER is the dissociative factor that is especially relevant to depression and anxiety, whereas ABS is the dissociative factor particularly relevant to OC symptoms. Indeed, DEP-DER was uniquely related to depression (Gómez-Pérez et al., 2013). They both share similar properties such as emotional numbing and feelings of detachment (Feeny et al., 2000); in addition, they are both associated with poor sleep (e.g., Breslau et al., 1996; Hayashino et al., 2010; for depression; and Giesbrecht et al., 2007 for dissociation). The specific link between anxiety and DEP-DER is possibly due to the hyperarousal and the hyperventilation that characterize anxiety and may produce DEP-DER (Sterlini and Bryant, 2002; Lickel et al., 2008); in addition, anxiety disorders are also related to impaired sleep (Ohayon and Roth, 2003; Gregory et al., 2005). Conversely, ABS seems to have a specific relation with OC symptoms (Soffer-Dudek, 2014, 2017a, 2018; Soffer-Dudek et al., 2015), possibly due to similar attentional mechanisms. OC symptoms include totally engaging one’s attention in internal obsessions such as imagined consequences of one’s behavior, or external stimuli in the form of compulsions. They are also characterized by a distrust of the senses, termed inferential confusion (O’Connor and Robillard, 1995). Similarly, ABS is characterized by narrowing awareness to one’s internal world (daydreaming) or external stimuli (e.g., a movie), with limited responsiveness to surroundings (Soffer-Dudek et al., 2015). Recently, it has been found that intensive absorption in daydreaming was related to OC symptoms (Soffer-Dudek and Somer, 2018).

### The Present Study

We hypothesized that depressive, anxiety, and OC symptoms may engender different links between the predictive momentary factors (sleepiness, current stress, and the subcomponents of rumination) and dissociative subscales. Specifically, we assumed that depression and anxiety would moderate the links between DEP-DER and its momentary predictors, so that these links would be stronger among participants characterized by high levels of trait anxiety or depression, than among participants characterized by low levels of anxiety or depression. Similarly, we hypothesized that OC symptoms would moderate the links of momentary predictors with ABS, so that stronger correlations would be found among participants with a high level of OC symptoms. It should be noted that we did not focus on the amnesia subcomponent of dissociation because it is less suitable for state measurements (Stiglmayr et al., 2009) and it has often been less useful in explorations of the specificity of relations of dissociation with psychopathological symptoms (e.g., Zucker et al., 2006; Simeon et al., 2007; Soffer-Dudek et al., 2015).

To conclude, the present study set out to: (1) examine if momentary feelings of sleepiness, current stress, and the subcomponents of ruminative thinking (negative value of the thought and thinking about the past) would predict state increased dissociation as assessed by specific scales: ABS and DEP-DER, using an experience sampling method; and (2) explore whether depression, anxiety, and OC symptoms play a role in moderating these links. We predicted that depression and anxiety would moderate links with DEP-DER and OC symptoms would moderate links with ABS.

## Materials and Methods

### Participants and Procedure

Ninety-nine undergraduate students (78.78% females, *M*age = 23.96, *SD*age = 1.97) participated in the study in exchange for 150 NIS (approximately $43). An additional two participants dropped out before the beginning of the study due to logistical or technical difficulties. Apart from age and gender, we assessed socioeconomic and familial status. We also asked whether they had ever taken part in psychotherapy (now or in the past), in order to gain a rough sense of the type of sample we have in relation to issues of mental health. The demographic characteristics of the sample are presented in Table 1.

**Table 1:** Demographic characteristics of the sample.

Variable	*N* = 99
Age *M* (*SD*)	23.96 (1.97)
Gender	
Male	*n* = 21 (21.21%)
Female	*n* = 78 (78.78%)
Marital status	
Unmarried	*n* = 97 (97.97%)
Married	*n* = 1 (1.01%)
Missing	*n* = 1 (1.01%)
Socio-economic status	
Low income family	*n* = 11 (11.11%)
Medium income family	*n* = 22 (22.22%)
High income family	*n* = 66 (66.66%)


*M, Mean; SD, standard deviation.*

The participants signed up for a study labeled “The relationship between patterns of sleep, thoughts, and mood” via the institutional psychological experiments system. First, participants received an explanation about study procedures during a 15-min session at the lab, during which they also signed a consent form, and downloaded the Personal Analytics Companion Application (PACO^1^) for the daily assessments. This application enables easy and accessible scheduled self-reports from participants’ mobile phones. After signing the consent form at the lab, during a time span of 2 days preceding the daily assessments participants completed trait questionnaires from their home computers, via online survey software (Qualtrics, Provo, UT, United States) in a single session (approximately 30-min). Next, they began the daily assessment phase, which took place on four consecutive weekdays. Four times a day, at fixed times (10:00, 13:30, 17:00, 20:30) the participants were alerted by the PACO application with a reminder to answer the state items. Thus, according to the study protocol, they were supposed to complete 16 experience-sampling assessment points overall; however, several participants completed either less or more assessment points on some of the days of the study, and some also continued their reports for a fifth day, resulting in a variable amount of overall assessment points (range 5–22, *M* = 16.03, *SD* = 2.62). On each such assessment, they completed the items via the application within approximately 5 min. Participants were instructed to respond to the questions as honestly as possible and to contact the researchers should they encounter any difficulties or concerns. The participants were subsequently debriefed regarding the purposes of the study. Notably, the participants also completed short sleep and dream questionnaires in the mornings, and four trait questionnaires (depression, stress, sleep, and dissociation) after completing the 4-day state assessment period. However, those measures are outside the scope of the current exploration. This study received ethical approval beforehand by the Ben-Gurion University institutional review board.

### Measures

#### Trait Measures

We refer to the following measures as trait measures because they are usually used to assess interpersonal differences, and in order to differentiate between them and momentary variables. Notably, however, the variables assessed by these questionnaires may not reflect stable characteristics of the individuals and rather reflect the level of these variables at the last period as they refer to the past weeks; still, they are assessed at the between-subjects level and thus we refer to them as traits. The first three measures (depression, anxiety, and OC symptoms) will be used in the main analyses of interest, whereas the rest of the trait measures will be used only for validating the state measures, described below. All measures have validated Hebrew versions.

##### Depression

The 16-item Quick Inventory of Depressive Symptomatology- Self-Report (QIDS-SR; Rush et al., 2003) measures nine components of depression (e.g., sad mood, sleep disturbance, interest) based on experiences in the past 7 days. The response scale ranges from 0 (e.g., “I do not feel sad”) to 3 (e.g., “I feel sad nearly all of the time”). The QIDS-SR has demonstrated reliability, validity, and sensitivity (Rush et al., 2003). Cronbach’s alpha in the present study was 0.71.

##### Anxiety

The 21-item Beck Anxiety Inventory (BAI; Beck et al., 1988) assesses anxiety symptoms on a 4-point scale (0- Not at all, to 3- Severely- it bothered me a lot). We modified the questionnaire from asking about the last 2 weeks to the last week in order to keep the depression and the anxiety questionnaires consistent. The BAI has demonstrated high internal consistency, test–retest reliability, and internal and external validity (Beck et al., 1988). Cronbach’s alpha in the present study was 0.86.

##### OC symptoms

The 18-item Obsessive–Compulsive Inventory-Revised (OCI-R; Foa et al., 2002) uses a 5-point Likert Scale (0- Not at all, 4- Extremely) that provides scores on six subscales (washing, checking, ordering, obsessing, hoarding, and neutralizing) and a total score. The OCI-R is a reliable and valid measure (Foa et al., 2002). Cronbach’s alpha in the present study was 0.91.

##### Stress

The 25-item Weekly Stress Inventory-Short Form (WSI- SF; Brantley et al., 2007) measures minor stress. The participants indicate whether various aversive events (e.g., “was ignored by others”) occurred to them during the past week, and the extent to which they were stressful, on a 7-point scale (0- Did not happen, 6- Very stressful). The WSI-SF was found to have good internal consistency and good convergent validity (Brantley et al., 2007). Cronbach’s alpha in the present study was 0.92.

##### Rumination

The 22-item Ruminative Response Scale (RRS) is a reliable and valid measure of rumination (Nolen-Hoeksema and Morrow, 1991). The items assess rumination on a 4-point scale (1- Almost never, 4- Almost always). According to Treynor et al. (2003), the RRS contains three subscales: depression, reflection, and brooding. The latter refers to passive and repetitive focus on the negative symptoms, which best suits the definition of rumination. Thus, we relied solely on the 5-item brooding factor as a measure of negative, passive and repetitive rumination. Cronbach’s alpha of the brooding scale in the present study was 0.78.

##### Worry

The 22-item Anxious Thoughts Inventory (AnTI; Wells, 1994) assesses worry in three basic dimensions: social worry, health worry, and meta-worry on a 4-point scale (1- Almost never, 4- Almost always). The AnTI possesses excellent psychometric properties (Wells, 1994). Cronbach’s alpha in the present study was 0.93.

##### Dissociation

The 28-item revised version of the Dissociative Experiences Scale (DES-II; Carlson and Putnam, 1993), measures the percentage of time that the individual experiences dissociation on an 11-point scale (0%- Never, 100%- Always). The three subscales of dissociation (ABS, amnesia, and DEP-DER) were computed based on a large-scale factor analysis including both psychiatric and non-clinical subjects (Carlson et al., 1991). The DES-II possesses excellent reliability and validity (Carlson and Putnam, 1993). Cronbach’s alpha in the present study was 0.89 for the total score, 0.84 for ABS, a somewhat low 0.65 for DEP-DER, and a low 0.56 for amnesia.

##### Sleep quality

The 19-item Pittsburgh Sleep Quality Index (PSQI; Buysse et al., 1989) assesses sleep quality during the past month. The PSQI possesses acceptable internal homogeneity, consistency, and validity (Buysse et al., 1989). The questionnaire includes seven components on a 4-point scale (0–3): subjective sleep quality, sleep latency, sleep duration, habitual sleep efficiency, sleep disturbances, use of sleeping medication and daytime dysfunction, as well as a total sleep quality score, on which we relied in the present study. Cronbach’s alpha in this study was 0.75.

#### State Measures

To examine the validity of state (momentary) measures, we wished to correlate their averages with trait measures. Thus, we calculated the mean score for each participant on each state variable, across all assessment points. We report the magnitude of these relationships below when describing each state measure. The correlations are Pearson product-moment coefficients with bootstrapped 95% confidence intervals (bias-corrected and accelerated).

##### Thinking about the Past

We used three items, based on Nezlek (2005), who measured daily rumination without the negative valence of the ruminative thinking (e.g., “How much today did you ruminate or dwell on things that happened to you?”). For the purposes of this study, instead of asking about today, we asked about the last few minutes. The participants scored these items on a 5-point scale (1- Not at all, 5- Extremely). The score for the “past” factor was calculated by averaging these three items. State thinking about the past (averaged across all measurements) and trait rumination (which includes the element of thinking about the past) were strongly correlated (*r* = 0.53 [0.33,0.70], *p* < 0.001).

##### Thinking about the Present

To parallel the past measure, we created three state items for thinking about the present, with an identical response scale. The three items of the “present” scale were: “In the last few minutes, to what extent…”: (a) “were you completely focused on the things that you were doing at the moment”; (b) “were your thoughts about the things that you were doing in the present” (c) “was your mind someplace else from your current occupation” (reversed item). The score for the present factor was calculated by averaging these three items.

##### Thinking about the Future

With the same response scale, we also created a “future” measure, based on the average of these three items: “In the last few minutes, to what extent…”: (a) “did you play back in your mind how you will act in a future situation”; (b) “did you dwell or ruminate on things that might happen in the future”; and (c) “did you dedicate time to re-think things that are expected to happen in the future.” State thinking about the future (averaged across all measurements) and trait worry (which includes the element of focusing on the future) were moderately correlated (*r* = 0.27 [0.08,0.46], *p* < 0.01).

##### Dissociation

State dissociation was assessed by a 4-item state scale taken from Soffer-Dudek (2017a), which was originally adapted from the Dissociation Tension Scale (DSS-4; Stiglmayr et al., 2003). The participants were asked, regarding the last few minutes, to what extent they got so involved in a daydream or in a movie/book that they were unaware of other events happening around them (ABS), and to what extent their body or the world felt strange or unusual (DEP and DER), on a 10-point scale (0 = Not at all, 9 = Very strongly). We combined the DEP and DER items to one DEP-DER score by averaging the two relevant items. However, the two items referring to ABS were not averaged to a single score, because we noticed that the items were uncorrelated with each other and exhibited different patterns of correlations with other items. We concluded that in hindsight, the absorption item that asked about a movie/book was less suitable for momentary experience-sampling because it was probably confounded with the number of actual movies or books that participants watched or read; thus, we decided not to use it, but rather to rely only on one item to assess state ABS (being involved in a daydream to the point of obliviousness to surroundings). The items of state DEP and DER were strongly correlated among themselves (when averaging state assessments across all measurements; *r* = 0.93, *p* < 0.001). Both DEP-DER and ABS state measurements, when averaged across all time points, were strongly correlated with their trait counterparts (*r* = 0.62 [0.36,0.80], *p* < 0.001, for DEP-DER; and *r* = 0.55 [0.28,0.75], *p* < 0.001 for ABS).

##### Momentary stress

The participants were asked to rate the extent to which they experienced stress in the last few minutes on a 5-point scale (1- Not at all, 5- Extremely) (Hellhammer and Schubert, 2012). State stress (averaged across all measurements) and trait stress were strongly correlated (*r* = 0.56 [0.40,0.70], *p* < 0.001).

##### Negative value of thoughts

The participants were probed about the emotional valence of their thought content (“In the last few minutes, the content of your thoughts was…”), rated on a 5-point symmetrical scale, in which a high score indicates negative valence (1- Positive, 5-Negative). This item, when averaged across all measurements, was moderately correlated with trait rumination (which includes focusing on negative content) (*r* = 0.31 [0.09,0.53], *p* < 0.005).

##### Sleepiness

We used the Stanford Sleepiness Scale (SSS; Hoddes et al., 1973), a valid and widely used method to measure state sleepiness with a single item. The participants were asked to rate their identification with one of 7 statements regarding the last few minutes, whereby a higher score indicates higher sleepiness (1- “Feeling active and vital; alert; wide awake,” 7- “Almost in reverie; sleep onset soon; lost struggle to remain awake”). State sleepiness averaged across all measurements was moderately correlated with trait poor sleep quality (*r* = 0.35 [0.14,0.55], *p* < 0.001).

### Data Analyses

The longitudinal design of the experience-sampling method produced a multilevel structure (Raudenbush et al., 2001; Singer and Willett, 2003). Thus, multilevel linear modeling (MLM) analysis was used. Level-1 data were the state measurements at each of the momentary assessment points. These Level-1 variables were nested within Level- 2 units, i.e., participants, characterized by demographic and trait data. We expected that Level-1 thinking about the past, and negative value of the thoughts (both representing components of rumination), sleepiness, and current stress, will predict Level-1 components of dissociation: ABS and DEP-DER. In each model, predicting either ABS or DEP-DER, we included one state predictor, and the measurement number variable (a variable ranging from 1 to a maximum of 22) in order to de-trend the model. We also hypothesized that there will be cross-level interactions, in that Level-1 predictive variables will interact with Level-2 trait psychopathology in predicting Level-1 dissociation. Specifically, we expected stronger effects of Level-1 predictors on dissociation under higher levels of baseline psychopathology.

We employed MLM using SPSS mixed models (version 24). The covariance structure type was AR(1), assuming stronger correlations between close measurements for each participant. Level-1 predictors (past, present, future, negative value, sleepiness, and current stress), and the variable representing time, were specified as both fixed and random effects, which means that their intercepts and slopes were allowed to vary among individuals. Level-2 depression, anxiety, and OC symptoms, and their interactions with Level-1 predictors, were specified only as fixed effects. Each model predicted either ABS or DEP-DER. All continuous predictors were grand-mean centered before conducting the analyses. Analyses of statistically significant interactions were performed based on Preacher et al. (2006), with high and low levels of trait variables defined as 1 SD above or below the sample mean, respectively. Standardized effect sizes for fixed effects rely on *semi-partial R^2^* statistics (Edwards et al., 2008).

## Results

Notably, MLM is a flexible method which is suitable for exploring linear associations even in the face of a variable number of repeated assessment points for each individual (Tabachnick and Fidell, 2007); there is no need to impute such missing data. However, other types of missing data (e.g., skipping items) should be evaluated and dealt with. Missing data patterns were estimated using the missing values analysis (MVA) function of SPSS (version 24). Missingness in this study varied between 0 to 2% for all variables. Little’s MCAR test was non-significant (χ*2* = 15.4, *p* = ns) suggesting that data were missing completely at random. The low percentage of the missing values and the random pattern of the missingness suggest that non-response in these data is ignorable (Tabachnick and Fidell, 2007). Histograms of all variables were inspected for detecting univariate outliers but there were none.

In order to examine the components of between-subjects and within-subjects variance for each Level-1 variable, we first computed intercept-only (null) models, in which no predictors were specified. The intraclass correlation (ICC) value reported below for these variables represents the proportion of between-subjects variance (i.e., the extent to which participants’ means vary from the general mean) out of the total variance (which also takes into account to what extent participants vary from their own mean). The ICC indices were 0.25, 0.33, 0.13, 0.33, 0.26, 0.45, 0.44, and 0.19 for past, future, present, negative valence, ABS, DEP-DER, current stress, and sleepiness, respectively. As these were all lower than 0.50, it may be concluded that all state measures varied more within the assessment points of each individual than between different individuals, suggesting that they are very much suitable to explore as state variables.

In Table 2, we present Pearson correlation coefficients, means and standard deviations of the trait variables (depression, anxiety, and OCD), and the state variables averaged across all momentary measurements (past, future, present, negative valence, current stress, sleepiness, ABS, and DEP-DER). As can be seen in the table, depression, anxiety, and OC symptoms were positively correlated with past, future, negative valence, current stress, and sleepiness, and inversely correlated with present.

**Table 2:** Correlations, means, and standard deviations of trait variables (depression, anxiety, OC symptoms), and state variables averaged across all measurements for each participant (thinking about the past, the future, and the present, negative value of thoughts, current stress, sleepiness, ABS, and DEP-DER).

	1	2	3	4	5	6	7	8	9	10	11
(1) Depression (*t*) (*M* = 4.98, *SD* = 3.37)	1	*0.57*^∗∗^	*0.59*^∗∗^	*0.38*^∗∗^	*0.30*^∗∗^	*-0.42*^∗∗^	*0.38*^∗∗^	*0.39*^∗∗^	*0.35*^∗∗^	*0.20*^∗^	*0.47*^∗∗^
		0.38,0.72	0.44,0.72	0.16,0.55	0.12,.47	-0.56,-0.24	0.18,0.59	0.20,0.55	0.15,0.51	-0.01,0.46	0.28,0.63
(2) Anxiety (*t*) (*M* = 6.49, *SD* = 6.00)		1	*0.49*^∗∗^	*0.53*^∗∗^	*0.49*^∗∗^	*0.40*^∗∗^	*0.36*^∗∗^	*0.57*^∗∗^	*-0.27*^∗∗^	*0.35*^∗∗^	*0.47*^∗∗^
			0.30,0.68	0.35,0.67	0.35,0.62	-0.54, -0.25	0.20,0.52	0.41, 0.69	0.06, 0.45	0.03,0.66	0.31,0.64
(3) OC symptoms *(t)* (*M = 11.98*, *SD = 9.42)*			1	*0.37*^∗∗^	*0.32*^∗∗^	*-0.33*^∗∗^	*0.28*^∗∗^	*0.37*^∗∗^	*0.34*^∗∗^	*0.20*^∗^	*0.57*^∗∗^
				0.15,0.55	0.12,0.52	-0.45,-0.21	0.09,0.45	0.18,0.54	0.17,0.48	-0.09,0.56	0.30,0.70
(4) Thinking about the past *(s)* (*M = 1.77, SD = 0.56)*				1	*0.79*^∗∗^	*-0.44*^∗∗^	*0.37*^∗∗^	*0.48*^∗∗^	*0.35*^∗∗^	0.18	*0.56*^∗∗^
					0.71,0.85	-0.57,-0.29	0.19,0.54	0.31,0.62	0.15,0.52	-0.11,0.56	0.35,0.72
(5) Thinking about the future *(s)* (*M = 2.26, SD = 0.74)*					1	*-0.49*^∗∗^	*0.35*^∗∗^	*0.57*^∗∗^	*-0.27*^∗∗^	0.11	*0.48*^∗∗^
						-0.64,-0.33	0.17,0.53	0.37,0.65	0.06,0.47	-0.10,0.41	0.30,0.63
(6) Thinking about the present *(s)* (*M* = 3.57, *SD* = 0.45)						1	*-0.58*^∗∗^	*-0.50*^∗∗^	*-0.50*^∗∗^	*-0.23*^∗^	*-0.46*^∗∗^
							-0.72,-0.44	-0.63,-0.33	-0.63,-0.34	-0.38,-0.15	-0.58,-0.34
(7) Negative value of the thoughts *(s)* (*M* = 2.41, *SD* = 0.6)							1	*0.46*^∗∗^	*0.47*^∗∗^	0.18	*0.30*^∗∗^
								0.31,0.64	0.21,0.70	0.05,0.32	0.08,0.51
(8) Current stress *(s)* (*M* = 1.82, *SD* = 0.69)								1	*0.36*^∗∗^	0.14	*0.24*^∗^
									0.15,0.55	-0.07,0.42	0.07,0.45
(9) Sleepiness *(s)* (*M* = 2.24, *SD = 59 )*									1	*0.24*^∗^	*0.34*^∗∗^
										0.10,0.40	0.14,0.52
(10) DEP-DER *(s)* (*M* = 1.12, *SD = 0.40)*										1	*0.41*^∗∗^
											0.16,0.60
(11) ABS *(s)* (*M* = 1.8, S*D* = 0.8)											1


*^∗^*p* < 0.05, ^∗∗^*p* < 0.01; Under each coefficient are 95% bootstrapped confidence intervals, using 1,000 resamples, calculated with the bias-corrected and accelerated method and rounded down to two decimals; Trait variables are marked by “*t”* and averaged state variables are marked by “*s”* in brackets.*

In Tables 3, 4, we present MLM models based only on Level-1 associations, and models depicting cross-level interactions, respectively. Specifically, associations between each of the state predictors (past, future, present, negative valence, current stress, and sleepiness) and each of the two predicted dissociation components (ABS and DEP-DER) are presented in Table 3, whereas the extent that these associations may be moderated by trait psychopathology variables can be seen in Table 4. Below, we describe the results for each predictor separately, and present results of simple slope analyses for statistically significant interactions. The full results of the simple slope analyses of the statistically significant interactions may be found in the Supplementary Material (see Supplementary Tables S1–S3, for depression, anxiety, and OCD, respectively).

**Table 3:** Results of multilevel models predicting either ABS or DEP-DER. Each model includes a Level-1 state predictor as well as the measurement number variable.

Predicted variable Predictor	*DEP-DER*	*ABS*
*Thinking about the past*	***b* = 0.04 [0.02,0.07], *SD* = 0.01, *t*(1055.97) = 3.09, *p* < 0.005 *R*^2^_β_ = 0.01**	***b* = 0.43 [0.36,0.50], S*D* = 0.03, *t*(1442.77) = 12.3, *p* < 0.001 *R*^2^_β_ = 0.10**
*Thinking about the future*	*b* = 0.01 [-0.01,0.04], *SD* = 0.01, *t*(875.71) = 1.02, *p* = ns *R*^2^_β_ = 0.00	***b* = 0.36 [0.30,0.42], *SD* = 0.03, *t*(1336.57) = 11.85, *p* < 0.001 *R*^2^_β_ = 0.10**
*Thinking about the present*	***b* = -0.04 [-0.06,-0.01], *SD* = 0.01, *t*(1341.24) = -2.87, *p* < 0.005 *R*^2^_β_ = 0.01**	***b* = -0.60 [-0.56,-0.54], *SD* = 0.03, *t*(1501.6) = -19.73, *p* < 0.001 *R*^2^_β_ = 0.21**
*Negative value of the thoughts*	***b* = 0.04 [0.01,0.07], *SD* = 0.01, *t*(1170.27) = 2.73, *p* < 0.01 *R*^2^_β_ = 0.01**	***b* = 0.22 [0.14,0.30], *SD* = 0.04, *t*(1449.25) = 5.76, *p* < 0.001 *R*^2^_β_ = 0.02**
*Current stress*	***b* = 0.04 [0.01,0.07], *SD* = 0.01, *t*(1102.6) = 2.74, *p* < 0.01 *R*^2^_β_ = 0.01**	***b* = 0.28 [0.21,0.36], *SD* = 0.04, *t*(1349.9) = 7.22, *p* < 0.001 *R*^2^_β_ = 0.04**
*Sleepiness*	***b* = 0.06 [0.04,0.08], *SD* = 0.01, *t*(1147.62) = 5.14, *p* < 0.001 *R*^2^_β_ = 0.02**	***b* = 0.27 [0.21,0.33], *SD* = 0.03, *t*(1418.97) = 8.89, *p* < 0.001 *R*^2^_β_ = 0.05**


*Statistically significant effects are bolded. Bootstrapping was performed using 1,000 resamples. CI = 95% bootstrapped confidence intervals, calculated with the bias-corrected and accelerated method and rounded down to two decimals.*

**Table 4:** Interactive models predicting ABS and DEP-DER, in which each of the Level-1 (state) predictors (thinking about the past, the future, the present, negative value of the thoughts, current stress, and sleepiness) is moderated by depression, anxiety, or OC symptoms.

The predicted variable:	*DEP-DER*				*ABS*	
		
Moderator predictor	Depression	Anxiety	OC symptoms	Depression	Anxiety	OC symptoms
*Thinking about the past*	***b* = 0.01 [0.01,0.02] *SD* = 0.00, *t*(329.72) = 3.89, *p* < 0.001 *R*^2^_β_ = 0.04**	***b* = 0.01 [0.01,0.01] *SD* = 0.00 *t*(99.31) = 6.2, *p* < 0.001 *R*^2^_β_ = 0.28**	***b* = 0.01 [0.00,0.01]^∗^*SD* = 0.00 *t*(415.66) = 3.69 *p* < 0.001 *R*^2^_β_ = 0.03**	***b* = 0.02 [0.01,0.04] *SD* = 0.01 *t*(1519.52) = 2.77, *p* < 0.01 *R*^2^_β_ = 0.01**	***b* = 0.01 [0.00,0.02]^∗^*SD* = 0.00 *t*(1536.02) = 2.34, *p* < 0.05 *R*^2^_β_ = 0.00**^†^	***b* = 0.01 [0.01,0.02]*SD* = 0.00 *t*(1535.23) = 4.01, *p* < 0.001 *R*^2^_β_ = 0.01**
*Thinking about the future*	*b* = 0.00 [0.00,0.01] *SD* = 0.00 *t*(495.44) = 0.55, *p* = 0.58 *R*^2^_β_ = 0.00	*b* = 0.00 [0.01,0.00] *SD* = 0.00 *t*(508.47) = -1.94, *p* = 0.053 *R*^2^_β_ = 0.01	*b* = 0.00 [0.00,0.00] *SD* = 0.00 *t*(524.53) = 0.32, *p* = 0.75 *R*^2^_β_ = 0.01	*b* = 0.01 [0.00,0.03] *SD* = 0.01 *t*(1512.39) = 1.7, *p* = 0.09 *R*^2^_β_ = 0.00	*b* = 0.01 [0.00,0.01] *SD* = 0.00 *t*(1506.22) = 1.47, *p* = 0.14 *R*^2^_β_ = 0.00	***b* = 0.01 [0.01,0.02] *SD* = 0.00 *t*(1494.54) = 4.22, *p* < 0.001 *R*^2^_β_ = 0.01**
*Thinking about the present*	*b* = 0.00 [0.01,0.01] *SD* = 0.00 *t*(1493.74) = -0.04 *p* = 0.96 *R*^2^_β_ = 0.00	*b* = 0.00 [-0.01,0.00] *SD* = 0.00 *t*(1497.7) = -1.4, *p* = 0.15 *R*^2^_β_ = 0.00	*b* = 0.00 [0.00,0.00] *SD* = 0.00 *t*(1463.72) = 0.06, *p* = 0.95 *R*^2^_β_ = 0.00	***b* = -0.04 [-0.06, -0.02] *SD* = 0.01 *t*(1534.28) = -4.34, *p* < 0.001 *R*^2^_β_ = 0.01**	***b* = -0.03 [-0.04,-0.02] *SD* = 0.00, *t*(1538.6) = -5.43, *p* < 0.001 *R*^2^_β_ = 0.02**	***b* = -0.02 [-0.02,-0.01] *SD* = 0.00 *t*(1499.55) = -5.15, *p* < 0.001 *R*^2^_β_ = 0.02**
*Negative value of the thought*	***b* = 0.01 [0.00,0.02]^∗^*SD* = 0.00 *t*(774.04) = 2.44, *p* < 0.05 *R*^2^_β_ = 0.01**	***b* = 0.01 [0.01,0.02] *SD* = 0.00 *t*(1204.2) = 4.69, *p* < 0.001 *R*^2^_β_ = 0.02**	***b* = 0.00 [0.00,0.01]^∗^*SD* = 0.00 *t*(590.88) = 1.99, *p* < 0.05 *R*^2^_β_ = 0.01**	***b* = 0.04 [0.02,0.06] *SD* = 0.01 *t*(1523.43) = 3.55, *p* < 0.001 *R*^2^_β_ = 0.01**	*b* = 0.00 [-0.01,0.01] *SD* = 0.01 *t*(1513.96) = -0.19, *p* = 0.85 *R*^2^_β_ = 0.00	***b* = 0.01 [0.01,0.02] *SD* = 0.00 *t*(1537.63) = 5.55, *p* < 0.001 *R*^2^_β_ = 0.02**
*Current stress*	***b* = 0.01 [0.00,0.02]^∗^*SD* = 0.00 *t*(1022.06) = 2.22 *p* < 0.05 *R*^2^_β_ = 0.01**	***b* = 0.01 [0.00,0.01]^∗^*SD* = 0.00 *t*(1028.28) = 2.25, *p* < 0.05 *R*^2^_β_ = 0.01**	*b* = 0.00 [0.00,0.01] *SD* = 0.00 *t*(987.99) = 1.56, *p* = 0.12 *R*^2^_β_ = 0.00	*b* = 0.01 [-0.01,0.04] *SD* = 0.01, *t*(1454.4) = 1.58, *p* = 0.11 *R*^2^_β_ = .00	*b* = -0.01 [-0.02,0.00] *SD* = 0.01 *t*(1455) = -1.52, *p* = 0.13 *R*^2^_β_ = 0.00	***b* = 0.01 [0.01,0.02] *SD* = 0.00 *t*(1511.84) = 4.22, *p* < 0.005 *R*^2^_β_ = 0.01**
*Sleepiness*	***b* = 0.01 [0.00,0.01]^∗^*SD* = 0.00 *t*(1512.98) = 2.32, *p* < 0.05 *R*^2^_β_ = 0.01**	***b* = 0.01 [0.01,0.01] *SD* = 0.00 *t*(1497.12) = 5.65, *p* < 0.001 *R*^2^_β_ = 0.02**	*b* = 0.00 [0.00,0.00] *SD* = 0.00 *t*(1490.7) = 1.65, *p* = 0.1 *R*^2^_β_ = 0.00	*b* = 0.00 [-0.02,0.02] *SD* = 0.00, *t*(1531.77) = 0.2, *p* = 0.84 *R*^2^_β_ = .00	*b* = 0.00 [-0.01,0.01] *SD* = 0.00 *t*(1540.67) = 0.44 *p* = 0.66 *R*^2^_β_ = 0.00	*b* = 0.00 [-0.01,0.00] *SD* = 0.00 *t*(1541) = -0.13, *p* = 0.89 *R*^2^_β_ = 0.00


*Statistically significant effects are bolded. Bootstrapping was performed using 1,000 resamples. CI = 95% bootstrapped confidence intervals, calculated with the bias-corrected and accelerated method and were rounded down to two decimals. ^∗^After the rounding, some of the lower borders of the significant effects that were positive, appear as if they are zero, when in fact they are positive but small. ^†^This effect was larger than zero but appears as zero when it is rounded down to two decimals.*

### Thinking About the Past, the Present, and the Future

Both ABS and DEP-DER were positively predicted by past and inversely predicted by present. Additionally, ABS was positively predicted by future. In addition to these main effects, several moderation effects emerged with all three psychopathology traits. Specifically, past was positively associated with DEP-DER only among those high in depression and OC symptoms (but not among those low in these variables) and the relation was significantly stronger among those with high levels of anxiety compared to those with low levels of anxiety. Similarly, past and present were more strongly associated with ABS among those high in psychopathology (depression, anxiety, or OC symptoms), compared to those low in psychopathology. Finally, the effect of thinking about the future on ABS was significantly stronger among participants with high levels of OC symptoms, compared to those with low levels.

### Negative Valence

Negative valence positively predicted DEP-DER and ABS. We were also interested in exploring the unique contribution of each one of the different ruminative thinking components to the prediction of dissociation scales; thus, we ran a combined model, in which we entered both past and negative valence as predictors in a single model. Each of these ruminative thinking elements remained statistically significant, both when predicting ABS (*b* = 0.40 [0.33,0.47], *SD* = 0.04, *t*(1462.38) = 11.22, *p* < 0.001, *R*^2^_β_ = 0.08, for past; *b* = 0.12 [0.04,0.19], *SD* = 0.04, *t*(1451.16) = 3.11, *p* < 0.005, *R*^2^_β_ = 0.03, for negative valence) and when predicting DEP-DER (*b* = 0.03 [0.01,0.06], *SD* = 0.01, *t*(941.62) = 2.44, *p* < 0.05, *R*^2^_β_ = 0.01, for past and *b* = 0.03 [0.01,0.06], *SD* = 0.01, *t*(1033.15) = 2.20, *p* < 0.0.05, *R*^2^_β_ = 0.01, for negative valence), suggesting that dissociation is explained uniquely and independently by each ruminative component.

In addition to these main effects, we found that the effect of negative valence on DEP-DER was present only among participants high in psychopathology (depression, anxiety, and OC symptoms). Similarly, the relation of negative valence with ABS was present only under high levels of depression and OC symptoms. Notably, an reviewer suggested that these findings may imply that negative valence and dissociation are not truly related, i.e., psychopathology may pose a confound. However, when examining the models, we found that the link between negative value and DEP-DER and the link between negative value and ABS remained statistically significant when depression, anxiety or OC symptoms were included in the model. These findings imply that the relationship between negative value and dissociative components probably cannot be explained by the confounding effect of psychopathology.

### Current Stress

Both DEP-DER and ABS were positively predicted by current stress. In addition to these main effects, different psychopathologies moderated these associations with DEP-DER and ABS. Specifically, the effect of current stress on DEP-DER was present only among those with high depression and anxiety, and the effect of current stress on ABS was present only among participants with high levels of OC symptoms.

### Sleepiness

Sleepiness positively predicted both DEP-DER and ABS. In addition to these main effects, we found that the effect of sleepiness on DEP-DER was present only among those high in depression and anxiety.

## Discussion

The main purpose of the study was to assess the extent to which hypothesized factors that fluctuate across different moments of the daily routine, specifically, current stress, sleepiness, and components of rumination (negative thinking and thinking about the past), may oscillate concurrently along with dissociative experiences, and whether symptoms of depression, anxiety, and OC symptoms moderate these associations.

First, it is interesting to note that rumination, dissociation, current stress, and sleepiness varied more within subjects than between subjects. Dissociation and rumination are often considered as trait characteristics, and individuals are described as “ruminative” or “dissociative.” It seems that although there are interpersonal differences in the inclination toward ruminative thinking or dissociation, as well as current stress or sleepiness, it is important to also explore these factors at the intrapersonal level, as state variables, as well as consider state-trait interactions as we did in the present study. Interestingly, trait and state relationships were not always completely parallel. Specifically, momentary increases in state DEP-DER were significantly related to negative value of the thought and thinking about the past; however, both negative valence of the thought and thinking about the past were unrelated to DEP-DER when examined as traits (i.e., averaged across all measurements). Apparently almost all of the participants experienced moments of thinking about the past or negative content at some point of the study. Hence, averaging across all the state measurements and ignoring momentary changes may result in missing some important effects and thus may be less informative. Similarly, van Heugten-van der Kloet et al. (2015a) have found that whereas dissociation and creativity were uncorrelated at the trait level, they were correlated at the state level. These results add precision to our understanding of relationships and thus highlight the importance of using person-centered designs, such as the present study.

### Current Stress, Sleepiness, Thought Components, and Dissociation

The findings support the suggested pathways between current stress, sleepiness and dissociative experiences, as both momentary stress and sleepiness predicted ABS and DEP-DER. Namely, when the participants reported that they felt sleepy or stressed, they were also more absorbed in daydreams and detached from the self or their surroundings. These findings reinforce a growing body of research regarding the relationship between sleep and dissociation (e.g., Giesbrecht et al., 2007; van der Kloet et al., 2012b; Soffer-Dudek et al., 2017). Additionally, they reinforce previous longitudinal (daily or experience-sampling) studies demonstrating that daily stress is temporally associated with dissociative experiences (Stiglmayr et al., 2008; Soffer-Dudek, 2017a). Notably, whereas the DEP-DER component is usually the one assessed in state dissociation questionnaires, our results suggest that being absorbed in daydreaming also relates contemporaneously to current stress and sleep. This replicates recent results regarding a contemporaneous relation of daily ABS with daily stress (Soffer-Dudek, 2017a), and expands them to an experience-sampling design, as well as to sleepiness. Indeed, ABS longitudinally predicted an increase in sleepiness in the face of sleep loss (Soffer-Dudek et al., 2017) and is related to post-traumatic stress (Armour et al., 2014) as well as psychopathological distress (Levin and Spei, 2004; Soffer-Dudek et al., 2015). These findings are also compatible with the recently formulated concept of MD (Somer, 2002); research on MD, which has been conceptualized as a disorder of extreme dissociative absorption, shows a relation with various comorbidities and psychopathological symptoms (Somer et al., 2017). Future studies may assess the extent to which the relation of current or daily stress and dissociative components may be the consequence of anteceding trauma; possibly the specific type of trauma is also of importance (i.e., type I- unanticipated single events, versus type II- repeated exposure to extreme events; Terr, 1995).

Regarding the components of negative thinking, we found that both elements of rumination, i.e., thinking about the past and negative value of the thought, significantly and independently predicted DEP-DER and ABS. Both components of worry, i.e., thinking about the future and negative value of the thought, predicted ABS, but only negative value predicted DEP-DER. In other words, it seems that when individuals thought either about the past or the future, they became absorbed in their inner world. However, a sense of detachment was associated only with thinking about the past. Possibly, negative events from the past that are unamenable to change (in comparison to future events), may bring about overwhelming sadness or guilt, and thus the need to detach from those feelings may be stronger. Moreover, DEP-DER may stem from intensely and vividly imagining a past or future situation, causing a person to feel disconnected from reality; it is possible that most people can conjure up such a vivid image more readily regarding past events that they have actually perceived, rather than imagined future situations. However, a reverse direction between DEP-DER and thinking about the past is also possible; for example, sensations of depersonalization or derealization may bring about thinking about the past in order to understand the origin of those sensations.

Although thinking about the past and the future differed in their links to DEP-DER, their relationships with the other variables were similar, whereas thinking about the present presented a completely opposite pattern. Specifically, both past and future were positively related to ABS, negative thinking, current stress, and sleepiness, whereas thinking about the present was inversely associated with all of these factors, as well as with DEP-DER. A closely related concept to thinking about the present, or rather to *not* thinking about the present, is mind-wandering. Mind-wandering is defined as unconstrained mental activity, generated by the individual rather than cued by an external environment (Smallwood and Schooler, 2006, 2015; Smallwood, 2013). Indeed, mind-wandering was strongly correlated with negative mood (Smallwood et al., 2009; Killingsworth and Gilbert, 2010; Poerio et al., 2013). Poerio et al. (2013) claim that mind-wandering, i.e., the trail of thought leaving external reality in favor of internally generated information, does not in itself lead to a deterioration in affect, but rather, the *affective content* of the wandering thought does. Indeed, our results show that thoughts about the present are often more positive than thoughts about the future or the past. However, thinking about the past predicted dissociative symptoms even when controlling for the negative valence of the thoughts, suggesting that this effect cannot be explained by affective content alone. Similarly, Smallwood and Schooler (2015) have noted that whereas poor mood has been related to mind-wandering in general, it is especially related to mind-wandering about the past. Importantly, despite the similarity between mind-wandering and dissociative ABS, Soffer-Dudek (2018) has found that these constructs are empirically disparate. Theoretically, dissociative ABS is defined by the intensity of focus on the stimuli (to the point of obliviousness to other stimuli), whereas mind-wandering is defined by the nature of the stimuli (internal rather than external; Soffer-Dudek, 2018).

Our findings are in accordance with the literature regarding the positive benefits of mindfulness meditation (e.g., Allen et al., 2006; Schonert-Reichl and Lawlor, 2010; Cullen, 2011). The core of the mindfulness approach is paying attention to the way that one’s experience occurs, without judging it (Kabat-Zinn, 2003). This practice aids in disengaging from mental states characterized by negative and ruminative thoughts and leads to the acceptance of mental contents (Morgan, 2003). In the present study, moments of focusing on the present were indeed related to decreased levels of momentary stress, sleepiness, negative thinking, and dissociative symptoms. Possibly, focusing on the present may prevent individuals from dissociating; this is in accordance with the usefulness of grounding techniques for coping with DEP-DER (Simeon, 2004), as well as the well-documented efficiency of mindfulness-based therapies for several psychopathological symptoms (Baer, 2003; Keng et al., 2011). Indeed, dissociation is detrimental to psychotherapy (Rufer et al., 2006; Spitzer et al., 2007; Fassino et al., 2009; Semiz et al., 2014; Arntz et al., 2015). Conversely, it is also possible that sleepiness, current stress and dissociative symptoms bring about thinking about the past or the future, possibly due to reduced concentration and thus difficulties in focusing on the present occupation.

### The Moderating Roles of Depression, Anxiety, and Obsessive–Compulsive Symptoms

In accordance with our expectation, the links described above were influenced by trait levels of psychopathology. Overall, we found support for our hypothesis that depression and anxiety would be more dominant in moderating the links with DEP-DER, whereas OC symptoms will demonstrate a stronger role in moderating the links with ABS. Specifically, we found that only depression and anxiety, but not OC symptoms, moderated the links between sleepiness and DEP-DER, so that moments of sleepiness were more strongly associated with moments of DEP-DER among those reporting high levels of depressive and anxiety symptoms. This may possibly be due to lower sleep quality in those with depression and anxiety (Breslau et al., 1996; Ohayon and Roth, 2003; Gregory et al., 2005; Hayashino et al., 2010), which possibly impairs the boundaries between sleep and waking states, resulting in DEP-DER, a dream-like waking state (Holmes et al., 2005). In addition, we found that current stress predicted DEP-DER only among individuals who were characterized by high levels of depression and anxiety. Anxiety, especially Panic Disorder, is characterized by hyperarousal (Clark and Watson, 1991; Joiner et al., 1999). Similarly, researchers studying depression from a physiologic standpoint have noted that individuals with depression have abnormally high levels of cortisol, similar to anxiety disorders (De Souza, 1995; Thase et al., 1996). This hyperarousal may possibly produce DEP-DER in order to counteract elevated arousal in an attempt to restore balance (Soffer-Dudek, 2014).

There seems to be a specific relation between ABS and OC symptoms (Soffer-Dudek, 2014). In accordance with our hypothesis, we found that OC symptoms moderated the links between thinking components and current stress on one hand, and with ABS on the other hand. For example, only individuals who were characterized by high levels of OC symptoms were inclined to report ABS alongside moments of current stress. Possibly, mental states such as thinking about the past or the future, negative content of thoughts, or current stress, triggered obsessive thoughts for high OC individuals, and during this process, they became absorbed in their inner world and detached from external stimuli. Conversely, becoming absorbed in thoughts or daydreams may have triggered anxiety when realizing their disconnection from reality, which may have induced the urge to check or perform another mental or behavioral compulsion.

### Limitations and Conclusion

There are several limitations in this study that should be addressed. First, all the measures were self-reported. Self-report measures may be vulnerable to bias, due to over-reporting (Frueh et al., 2000; Merckelbach et al., 2014). However, over-reporting bias was found to be relevant only to 4% of participants in studies with no secondary gain (Merckelbach et al., 2014). Moreover, over-reporting often decreases effect sizes rather than increases them (Merckelbach et al., 2014) and the extent to which it distorts research outcomes is limited (McGrath et al., 2010). Finally, individual differences in the tendency to over-report are irrelevant to within-subjects variance, which was the main focus of the present investigation. Thus, our pattern of results probably cannot be fully explained by over-reported symptoms of the participants.

It is important to state that we did not assess trauma in this study. The link between trauma and dissociation has been extensively explored and trauma is considered to be one of the main factors predicting dissociation (e.g., Gershuny and Thayer, 1999). Assessing past traumas in this sample could have promoted a better understanding of the possible interactions between long-term and short-term correlates of dissociation. In addition, in the present study we explored the structure components of rumination (past focus and negative valence of the repetitive thought) but we did not explore the contents or themes embodied in the ruminative thoughts. For example, Sar and Ozturk (2006) suggested that past unresolved traumatic events lead to repetitive obsessions about these traumas. Further research is needed in order to explore whether the effect of rumination in predicting dissociation is related to specific (e.g., traumatic) contents or rather if it is independent of any specific content, stemming only from to the structure of this thought style.

The participants were tested only for 4 days. The short duration of the study was was determined in order to recruit a larger sample by making the study more accessibe and less demanding. In these 4 days, the participants answered the items four times a day, resulting in 16 assessment points. This resulted in a sufficient amount of within subjects’ variance as can be concluded from the ICC indices and also from the strong within-subjects associations that were found. Yet, longer study with more days of measurment could have increased the validity of our findings.

Another limitation is the limited number of items assessing state ABS and DEP-DER. ABS was measured with only one item, due to a problem with the validation of the second item. One item is not fully sufficient to assess all the aspects of ABS and this item indeed measured only absorption in day-dreaming (and not in other mental or external experiences). DEP-DER was measured with two items, one for DEP and one for DER, which is also insufficient. According to Sar et al. (2017), there are four aspects of DEP-DER: cognitive-emotional depersonalization, perceptual alterations, detachment from reality, and bodily self-detachment. Of these four aspects, our two items measured cognitive-emotional depersonalization and detachment from reality, whereas the other two aspects were not examined in our study. The nature of our study (experience sampling) limited the amount of items, in order to avoid overloading each administration point. In future studies, more items may be used in order to validate and to expand our results to additional aspects of both ABS and DEP-DER.

A final limitation of our study is the fact that our study was gender-biased toward women and was composed of non-clinical and relatively high-functioning college students. However, the definition of the sample as non-clinical does not mean that none of the participants had clinical-range symptoms and disorders. In fact, 40% of participants reported that they had, at some point in their life, engaged in psychotherapy, and 10% of them reported that they currently take psychotropic medication. These percentages are compatible with reports by Hunt and Eisenberg (2010) and Stallman (2010) who claim that psychopathology levels among college students are high and similar to the prevalence of psychopathology in same-aged non-students. The non-clinical nature of the sample in this study, may explain the low means of DEP-DER and ABS state scales and the low means of depression and anxiety trait scales. The ICC indices of DEP-DER and ABS indicate that there was sufficient amount of within-subject variance to explore temporal changes in these variables. The means of depression and anxiety are completely compatible with the means of non-clinical subjects in other studies (e.g., Crawford et al., 2011 for anxiety; and Gonzalez et al., 2013 for depression). Thus, we may be able to generalize our results to community samples, but future research should explore these links among clinical samples with higher levels of dissociation, as well as depression and anxiety.

Despite these limitations, the study also has methodological strengths, specifically, the use of experience-sampling to identify high-resolution contemporaneous relationships, and the exploration of cross-level trait-state interactions which help advance scientific knowledge beyond simple correlative information. There are some important findings in this study. First, whereas most studies on the mechanisms which may lead to dissociative experiences are cross-sectional, in this study we used momentary assessments and expanded previous results regarding momentary stress and dissociation (Stiglmayr et al., 2008) to sleepiness and rumination. Second, we broke apart the concepts of rumination and worry that are usually examined in the literature as whole concepts, into their basic thought components, and showed that each component has a unique and significant role in predicting dissociation. Specifically, thinking about the past, the future, and negative value of the thoughts, are all unique predictors of state dissociative experiences. Third, we found that thinking about the past or the future are both related to negative mental states (current stress, sleepiness, and dissociation), whereas thinking about the present was associated with decreased levels of those states. However, it is important to take into account that (a) our data points to shared oscillations between the constructs, but it does not identify the directionality of the associations; and (b) these links did not exist among all participants equally, but rather were dependent upon levels of psychopathological symptoms.

These findings expand our knowledge on the mechanisms involved in dissociative experiences, and they have implications for clinical practice. Specifically, clinicians treating common disorders such as depression, anxiety, and OCD, do not necessarily routinely assess dissociation; however, our findings highlight the importance of taking dissociation into account. Dissociation is maladaptive and related to poorer prognosis and poorer response to treatment, possibly due to detachment from the therapeutic process (Rufer et al., 2006; Spitzer et al., 2007; Fassino et al., 2009; Semiz et al., 2014; Arntz et al., 2015). Thus, it is especially important to identify the mental states that may trigger dissociation in psychotherapy. Perhaps clinicians who observe signs of repetitive thinking about events from the past or the future should pay attention to possible detachment accompanying those moments. Such dissociating may be detrimental to the therapeutic process if it goes unnoticed and untreated. Moreover, our findings suggest that clinicians may expect specific subtypes of dissociation in accordance with the psychopathological pattern of the client. Such expansion of our knowledge on the links between dissociation and psychopathology may be helpful in early recognition of dissociation signs, ultimately leading to better understanding clients’ emotional states across different moments in the session.

## Data Availability Statement

The raw data supporting the conclusions of this manuscript will be made available by the authors, without undue reservation, to any qualified researcher.

## Ethics Statement

This study was carried out in accordance with the recommendations of the BGU institutional review board with written informed consent from all subjects. All subjects gave written informed consent in accordance with the Declaration of Helsinki. The protocol received ethical approval beforehand by the institutional review board.

## Author Contributions

MV-L and NS-D were responsible for the conception, literature review, data collection, data analysis, and writing of this manuscript. This research is part of MV-L’s graduate work, conducted under the supervision of NS-D.

## Conflict of Interest Statement

The authors declare that the research was conducted in the absence of any commercial or financial relationships that could be construed as a potential conflict of interest.

## Footnotes

1 http://pacoapp.com
