# Supplementary material for: No Time Like the Present: Thinking About the Past and the Future Is Related to State Dissociation Among Individuals With High Levels of Psychopathological Symptoms
Source: Front Psychol. 2018 Dec 7;9:2465. doi: 10.3389/fpsyg.2018.02465 (PMC6292924; doi:10.3389/fpsyg.2018.02465)
Supplement: Supplementary file 1 [file Table_1.DOCX]

Supplementary Material

**No time like the present: thinking about the past and the future is related to state dissociation among individuals with high levels of psychopathological symptoms.**

**Miriam Vannikov-Lugassi^1*^ and Nirit Soffer-Dudek**

*** Correspondence:** Miriam Vannikov-Lugassi, miriamv@post.bgu.ac.il

Table S1

The simple slopes of thinking about the past, the future, the present, negative value of the thoughts, current stress, and sleepiness on DEP-DER and ABS under low and high depression levels.

| *ABS* | | *DEP-DER* | | The predicted variable |
| --- | --- | --- | --- | --- |
| High depression | Low depression | High depression | Low depression | Level of depression  The predictor |
| ***b*=.47 [.39, .55]**  ***SD=*.04**  ***t*(1462.99)=**  **11.34 *p*<.001**  ***R*^2^*_β_* = .18** | ***b*=.30 [.20, .40]**  ***SD=*.05**  ***t*(1501.15)=**  **5.97 *p*<.001**  ***R*^2^*_β_* = .05** | ***b*=.08 [.04, .11]**  ***SD=*.02**  ***t*(609.49)=**  **4.53 *p*<.001**  ***R*^2^*_β_* = .07** | *b*= -.02 [-.06, .02] *SD=*.02  *t*(837.5)=  -.81 *p*= .41  *R*^2^*_β_* = .00 | *Thinking about the past* |
| ***-*** | - | *-* | - | *Thinking about the future* |
| ***b*= -.72 [-.81, -.64] *SD=*.04**  ***t*(1540.59)=**  **-16.52 *p*<.001**  ***R*^2^*_β_* = .35** | ***b*= -.46 [-.54, -.38] *SD=*.04**  ***t*(1536.52)=**  **-10.89 *p*<.001**  ***R*^2^*_β_* = .15** | *-* | - | *Thinking about the present* |
| ***b*=.31 [.20, .42]**  ***SD=*.05**  ***t*(1527.88)=**  **5.68 *p*<.001**  ***R*^2^*_β_* = .04** | *b*=.04 [-.06, .15]  *SD=*.05  *t*(1436.26)=  .85 *p*=.39  *R*^2^*_β_* = .00 | ***b*=.07 [.03, .11]**  ***SD=*.02**  ***t*(996.97)=**  **3.27 *p*<.005**  ***R*^2^*_β_* = .02** | *b*= .00 [-.04, .04] *SD=*.02  *t*(1040.41)=  -.09 *p*=.92  *R*^2^*_β_* = .00 | *Negative value of the thought* |
| *-* | - | ***b*=.06 [.02, .10]**  ***SD=*.02**  ***t*(1043.87)=**  **3.05 *p*<.005**  ***R*^2^*_β_* = .02** | *b*= -.00 [-.04, .04] *SD=*.02  *t*(1150.73)=  -.17 *p*=.86  *R*^2^*_β_* = .00 | *Current stress* |
| *-* | - | ***b*=.08 [.05, .11]**  ***SD=*.01**  ***t*(1450.55)=**  **5.17 *p*<.001**  ***R*^2^*_β_* = .04** | *b*=.03 [.00, .06]  *SD=*.02  *t*(1448.41)=  1.86 *p*=.06  *R*^2^*_β_* = .00 | *Sleepiness* |

*Note.* The simple slope analyses were performed only for variables that were significantly moderated by depression according to the interactive models; Statistically significant slopes are bolded. Bootstrapping was performed using 1,000 resamples. CI = 95% bootstrapped confidence intervals, calculated with the bias-corrected and accelerated method and were rounded down to two decimals.

Table S2

The simple slopes of thinking about the past, the future, the present, negative value of the thought, current stress, and sleepiness on DEP-DER and ABS under low and high anxiety levels.

| *ABS* | | *DEP-DER* | | The predicted variable |
| --- | --- | --- | --- | --- |
| High anxiety | Low anxiety | High anxiety | Low anxiety | Level of anxiety  The predictor |
| ***b*=.45 [.37, .54]**  ***SD=*.04**  ***t*(1516.58) =**  **10.86 *p*<.001**  ***R*^2^*_β_* = .07** | ***b*=.32 [.22, .42]**  ***SD=*.05**  ***t*(1506.84) =**  **6.43 *p*<.001**  ***R*^2^*_β_* = .03** | ***b*=.09 [.06, .12]**  ***SD=*.02**  ***t*(343.22) =**  **5.4 *p*<.001**  ***R*^2^*_β_* = .08** | ***b*= -.05 [-.08, -.01] *SD=*.02**  ***t*(382.25) =**  **-2.45 *p*<.05**  ***R*^2^*_β_* = .01** | *Thinking about the past* |
| *-* | - | *-* | - | *Thinking about the future* |
| ***b*= -.75 [-.83, -.66] *SD=*.04**  ***t*(1540.65) =**  **-17.67 *p*<.001**  ***R*^2^*_β_* = .17** | ***b*= -.43 [-.51, -.34] *SD=*.04**  ***t*(1529.97) =**  **-10.09 *p*<.001**  ***R*^2^*_β_* = .06** | *-* | - | *Thinking about the present* |
| *-* | - | ***b*=.10 [.06, .15]**  ***SD=*.02**  ***t*(1313.75) =**  **4.87 *p*<.001**  ***R*^2^*_β_* = .02** | *b*= -.03 [-.07, .01] *SD=*.02  *t*(1150.54) =  -1.7 *p*=.09  *R*^2^*_β_* = .00 | *Negative value of the thoughts* |
| *-* | - | ***b*=.04 [.01, .08]**  ***SD=*.02**  ***t*(1072. 2)=**  **2.24 *p*<.05**  ***R*^2^*_β_* = .01** | *b*= -.02 [-.06, .02] *SD=*.02  *t*(1180.36)=  -.86 *p*=.38  *R*^2^*_β_* = .00 | *Current stress* |
| *-* | - | ***b*=.10 [.07, .13]**  ***SD=*.01**  ***t*(1435.55)=**  **7.21 *p*<.001**  ***R*^2^*_β_* = .03** | *b*= .00 [-.03, .03] *SD=*.02  *t*(1449.65)=  -.23 *p*=.81  *R*^2^*_β_* = .00 | *Sleepiness* |

*Note.* The simple slope analyses were performed only for variables that were significantly moderated by anxiety in the interactive models. Statistically significant slopes are bolded. Bootstrapping was performed using 1,000 resamples. CI = 95% bootstrapped confidence intervals, calculated with the bias-corrected and accelerated method and were rounded down to two decimals.

Table S3

The simple slopes of thinking about the past, the future, the present, negative value of the thought, Current stress and sleepiness on DEP-DER and ABS under low and high levels of OC symptoms.

| *ABS* | | *DEP-DER* | | The predicted variable |
| --- | --- | --- | --- | --- |
| High OCD | Low OCD | High OCD | Low OCD | Level of OCD  The predictor |
| ***b*=.51 [.43, .60]**  ***SD=*.04**  ***t*(1471.61)=**  **11.57 *p*<.001**  ***R*^2^*_β_* = .08** | ***b*=.26 [.16, .36]**  ***SD=*.05**  ***t*(1520.86) =**  **5.33 *p*<.001**  ***R*^2^*_β_* = .02** | ***b*=.08 [.04, .12]**  ***SD=*.02**  ***t*(724.55) =**  **4.45 *p* <.001**  ***R*^2^*_β_* = .03** | *b*= -.01 [-.05, .03] *SD=*.02  *t*(815.06) =  -.64 *p* =.51  *R*^2^*_β_* = .00 | *Thinking about the past* |
| ***b*=.45 [.37, .54]**  ***SD=*.04**  ***t*(1376.58)=**  **10.7 *p*<.001**  ***R*^2^*_β_* = .08** | ***b*=.20 [.12, .28]**  ***SD=*.04**  ***t*(1461.51) =**  **4.92 *p*<.001**  ***R*^2^*_β_* = .02** | *-* | - | *Thinking about the future* |
| ***b*=-.74 [-.83, -.66]**  ***SD=*.04**  ***t*(1538.3)=**  **-17.05 *p*<.001**  ***R*^2^*_β_* = .16** | ***b*= -.43 [-.51, -.35] *SD=*.04**  ***t*(1540.36) =**  **-10.36 *p*<.001**  ***R*^2^*_β_* = .06** | *-* | - | *Thinking about the present* |
| ***b*=.29 [.19, .40]**  ***SD=*.05**  ***t*(1516.28)=**  **5.56 *p*<.001**  ***R*^2^*_β_* = .02** | *b*=.06 [-.04, .16]  *SD=*.05  *t*(1475.56) =  1.24 *p*=.21  *R*^2^*_β_* = .00 | ***b*=.06 [.02, .10]**  ***SD=*.02**  ***t*(859.67) =**  **2.9 *p*<.005**  ***R*^2^*_β_* = .01** | *b*=.01 [-.03, .04]  *SD=*.02  *t*(980.27)=  .29 *p* =.77  *R*^2^*_β_* = .00 | *Negative value of the thoughts* |
| ***b*=.32 [.22, .41]**  ***SD=*.05**  ***t*(1421.75)=**  **6.36 *p*<.001**  ***R*^2^*_β_* = .03** | *b*=.09 [-.02, .20]  *SD=*.06  *t*(1443.17) =  1.65 *p*=.1  *R*^2^*_β_* = .00 | *-* | *-* | *Current stress* |
| *-* | - | *-* | *-* | *Sleepiness* |

*Note.* The simple slope analyses were performed only for variables that were moderated by OC symptoms in the interaction's analyses; Statistically significant effects are marked by bold letters. Bootstrapping was performed using 1,000 resamples. CI = 95% bootstrapped confidence intervals, calculated with the bias-corrected and accelerated method and were rounded down to two decimals.
